# Formation of highly toxic hydrogen cyanide upon ruby laser irradiation of the tattoo pigment phthalocyanine blue

**DOI:** 10.1038/srep12915

**Published:** 2015-08-05

**Authors:** Ines Schreiver, Christoph Hutzler, Peter Laux, Hans-Peter Berlien, Andreas Luch

**Affiliations:** 1 German Federal Institute for Risk Assessment (BfR), Department of Chemical and Product Safety, Max-Dohrn-Strasse 8-10, 10589 Berlin, Germany; 2 Evangelical Elisabeth Hospital, Department of Laser Medicine, Lützowstrasse 24-26, 10785 Berlin, Germany

## Abstract

Since laser treatment of tattoos is the favored method for the removing of no longer wanted permanent skin paintings, analytical, biokinetics and toxicological data on the fragmentation pattern of commonly used pigments are urgently required for health safety reasons. Applying dynamic headspace—gas chromatography with mass spectrometric detection (DHS—GC/MS) and comprehensive two-dimensional gas chromatography coupled to time-of-flight mass spectrometry (GCxGC—ToF-MS), we identified 1,2-benzene dicarbonitrile, benzonitrile, benzene, and the poisonous gas hydrogen cyanide (HCN) as main fragmentation products emerging dose-dependently upon ruby laser irradiation of the popular blue pigment copper phthalocyanine in suspension. Skin cell viability was found to be significantly compromised at cyanide levels of ≥1 mM liberated during ruby laser irradiation of >1.5 mg/ml phthalocyanine blue. Further, for the first time we introduce pyrolysis-GC/MS as method suitable to simulate pigment fragmentation that may occur spontaneously or during laser removal of organic pigments in the living skin of tattooed people. According to the literature such regular tattoos hold up to 9 mg pigment/cm^2^ skin.

Inserting inorganic or organic pigments into the dermis for the purpose of tattooing or permanent make-up represents an unbroken modern trend world-wide^1^,^2^. At the same time, dramatically increasing numbers of tattooed people result in a similarly increasing demand for laser removal of tattoo pictures and designs that occasionally become subject of embarrassment and deepest regret later in life^1^,^3^. In this regard the potential risk for pigment cleavage into toxic or carcinogenic fragments upon tattoo laser treatment or even just during the exposure of skin to regular light (e.g. while sunbathing) are increasingly recognized as a serious health-related issue^1^,^4^,^5^,^6^,^7^. Due to insufficient research, the identity of the possible chemical descendants and their long term effects when being released into systemic distribution throughout the human body remains unclear. Data on laser decomposition of pigments used in tattoo inks are available for a few azo dyes only^5^,^6^,^7^,^8^. On the other hand, there is a complete lack of data that would look into the decomposition and fate of rather lightfast molecule species such as phthalocyanines, in particular when it comes to the irradiation of cutaneous pigment deposits with medical lasers.

To the best of our knowledge copper phthalocyanine (also called phthalocyanine blue or pigment B15:3) is the only blue organic pigment currently used in tattoo inks on the European market^9^. Yet data regarding its safety as tattoo pigment and its decomposition behavior are presently not available. At the least, a most recent review cited two unpublished studies (PhD theses) that failed to detect any fragmentation of highly lightfast pigments including copper phthalocyanine upon irradiation with ultraviolet (UV) or visible (VIS) light^10^. In contrast to the well-known UV/VIS-induced photolysis of azo dyes, color fading upon laser irradiation of tattoo pigments is therefore assumed to be rather the result of heat-dependent chemical decomposition (photothermolysis) and the breakup of particles followed by cellular clearance (i.e. phagocytosis by macrophages)^6^. Since laser irradiation of tattoo pigments can produce local temperatures exceeding 1,000 °C, the fracturing of the particles by steam-carbon reactions will be induced^11^,^12^. All of these assumptions are supported by the observation that only empty tissue vacuoles can be found in upper dermal layers directly after laser irradiation, whereas smaller fragmented particles remain in the mid dermal layers accompanied by mild infiltration of macrophages^13^. Hence chemical decomposition toward atomization is thought to be the main cause of pigment fading in laser treatments^13^.

In clinical dermatology amongst others ruby lasers are commonly used for the irradiation of colored tattoos^14^. Since they are particularly effective in the treatment of blue pigments, marked heat-dependent decomposition is likely to occur. To achieve the required high temperatures selectively inside the pigment deposits and—at the same time—preventing any major heat transfer to the surrounding tissue, short pulsed q-switched or the newly developed picosecond lasers are now common practice^15^,^16^.

In our study we applied pyrolysis conditions to mimic the laser-induced and temperature-dependent decomposition of the blue pigment copper phthalocyanine. Online coupling to gas chromatographic (GC) separation of the occurring fragmentation products and subsequent mass spectrometric (MS) analyses enabled us to identify all emerging volatile descendants based on available mass spectra libraries. To prove the relevance of the detected decomposition product pattern of copper phthalocyanine, we conducted q-switched ruby and neodymium-doped yttrium aluminium garnet (Nd:YAG) laser irradiation experiments, the latter of which is also used in dermatology for the purpose of tattoo removal^14^. Since pyrolysis–GC/MS (Py-GC/MS) analyses also provided evidence for the occurrence of very volatile and highly toxic compounds such as hydrogen cyanide (HCN) and benzene, we further developed a dynamic headspace (DHS) method to avoid any significant loss of the volatile compounds during work-up and analysis. By applying DHS along with two-dimensional gas chromatography coupled to time-of-flight mass spectrometry (GCxGC—ToF-MS) we were then able to sensitively and specifically address all of the expected compounds irrespective of their tendency to vaporize.

The detection and unambiguous identification of the highly toxic gas HCN further prompted us to investigate whether its level released via laser decomposition of copper phthalocyanine would be high enough to induce cellular toxicity. To this end we applied cyanide (CN^–^) concentrations that corresponded to the range of HCN released upon laser cleavage of pigment B15:3 and measured the inhibition of the mitochondrial electron transport chain via declining ATP levels in HaCaT cells *in vitro.* In summary the present work is the first that proves the occurrence of hazardous compounds upon laser-induced decomposition of the lightfast tattoo pigment copper phthalocyanine at concentrations high enough to produce cellular harm.

## Results

### Pyrolysis-GC/MS of copper phthalocyanine blue

**Figure 1:**
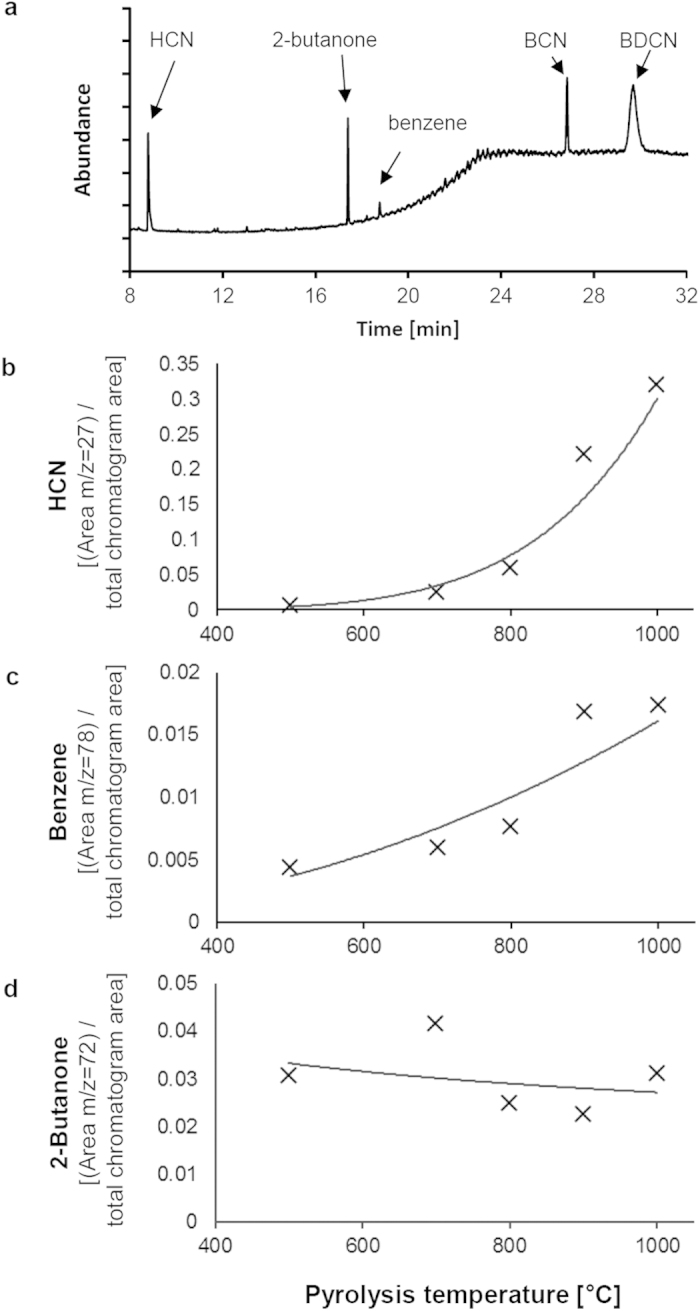
Phthalocyanine blue (pigment B15:3) is cleaved into BDCN, BCN and HCN upon pyrolysis. (**a**) Py–GC/MS chromatogram (1,000 °C) of pigment B15:3. BDCN, BCN, HCN, and 2-butanone represent the main compounds detected. Some tiny amounts of benzene were also detectable at temperatures >800 °C. (**b**)–(**c**) Temperature-dependent formation of HCN and benzene during Py–GC/MS analysis of the pigment phthalocyanine blue (pigment B15:3). The values shown represent extracted ion areas of (**b**) HCN (m/z = 27), (**c**) benzene (m/z = 78), and (**d**) 2-butanone (m/z = 72) that have been normalized to the total chromatogram area. Therefore, absolute numbers do not correlate to peak areas of all ion fragments of the molecules included (cf. Fig. 1a). Similar as with BCN and BDCN (data not shown), HCN and benzene formation increases with pyrolysis temperature while levels of 2-butanone did not.

**Figure 2:**
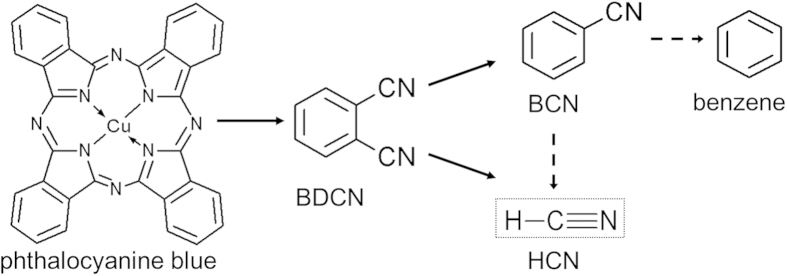
Decomposition pattern of phthalocyanine blue (pigment B15:3) based on the pyrolysis and laser irradiation data presented.

Using an online coupling to GC/MS we identified HCN, 1,2-benzene dicarbonitrile (BDCN), benzonitrile (BCN), and 2-butanone as the four main cleavage products of pigment B15:3 upon pyrolysis at ≥800 °C (Fig. 1a). Since the latter is often added as solvent its presence is likely a remainder from pigment synthesis. In addition, some traces of benzene also emerged in the pyrograms at temperatures >800 °C. In contrast to 2-butanone, levels of HCN, BDCN, BCN and benzene were increasing with pyrolysis temperature, thus confirming their occurrence as specific degradation products (Fig. 1b–d). Commercially available standards for HCN (including its isotopes) and BDCN were used for the identification of pyrolysis-dependent descendants of the pigment. Further, retention times and mass spectra were identical to those peaks identified as HCN and BDCN by library comparison. Whereas the decomposition of pigment B15:3 into BDCN has been already shown by a previous study^17^, additional formation of the lower molecular weight compounds BCN and benzene, and the gaseous HCN has remained undiscovered yet (Fig. 2).

### Laser irradiation of copper phthalocyanine blue

**Figure 3:**
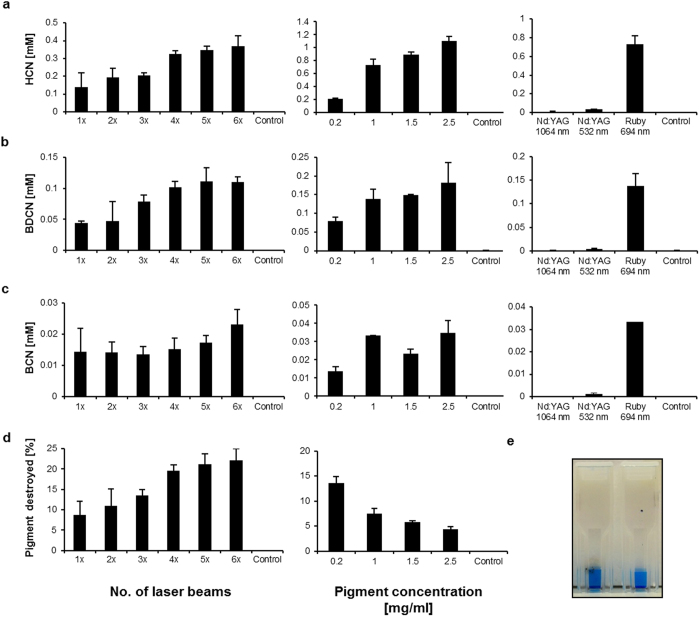
Phthalocyanine blue (pigment B15:3) is cleaved into BDCN, BCN and HCN upon laser irradiation. (**a**)–(**c**) left: Levels of BDCN, BCN and HCN depending on the number of applied ruby laser pulses (1× to 6×; initial pigment concentration used: 0.2 mg/ml; control = no laser beam). (**a**)–(**c**) middle: BDCN, BCN and HCN levels as function of the initial pigment concentration (0.2 mg/ml to 2.5 mg/ml; at each concentration 3 laser pulses applied; control = no pigment). (**a**)–(**c**) right: Only slightly increased fragment concentrations were found upon Nd:YAG laser irradiation when compared to ruby laser irradiation (3 laser pulses at 1 mg/ml pigment; control = no laser beam). (**d**) left: Fraction (in %) of pigment destroyed depending on the number of ruby laser pulses applied (1× to 6×; initial pigment concentration used: 0.2 mg/ml; control = no laser beam). (**d**) right: Fraction (in %) of pigment destroyed depending on the initial pigment concentration (0.2 mg/ml to 2.5 mg/ml; at each concentration 3 laser pulses applied; control = no pigment). (**e**) UV/VIS cuvette after 3 laser pulses (left) compared to an untreated sample (right) containing 0.2 mg/ml of pigment B15:3 each. The laser-treated sample is carbonized at the outer cuvette surface but appears more intensely blue colored (see text for further details). All values are displayed as mean ± SD (n = 3).

To investigate whether the decomposition patterns are similar under various laser treatment scenarios applied in cosmetic dermatology, water-based pigment dispersions of pigment B15:3 were either treated with multiple quantities of ruby (5 J/cm^2^, spot size 5 mm, 694 nm) or Nd:YAG (5 J/cm^2^, spot size 3 mm, at 1,064 nm or 532 nm) laser pulses. Subsequent to laser irradiations quantitative analysis of both volatiles HCN and benzene was carried out using a DHS–GC/MS method, whereas screening and quantification of the other fragments (i.e. BDCN, BCN) were carried out by extraction with ethyl acetate followed by GCxGC-ToF-MS analysis. So, HCN, BCN and BDCN levels were all found increasing as a function of the number of ruby laser beams applied and the initial pigment concentrations (Fig. 3a–c). On the other hand, quantitative analysis of benzene revealed kind of challenging due to its occurrence only in traces and the necessity for an adaptation of the analytics applied (which were intentionally optimized to target CN-containing compounds). Nevertheless we were able to detect 0.32 μM (25.1 ng/ml) benzene after three ruby laser irradiation pulses applied to 1 mg pigment B15:3 per ml suspension (mean out of n = 3). With this we experimentally confirmed the occurrence of all intermediates in the degradation pathway of phthalocyanine blue according to theoretical and reasonable predictions (Fig. 2). We further demonstrated that the decomposition products in aqueous suspensions of pigment copper phthalocyanine upon ruby laser irradiation correlate well with those found in the corresponding Py-GC/MS analyses (cf. above).

**Figure 4:**
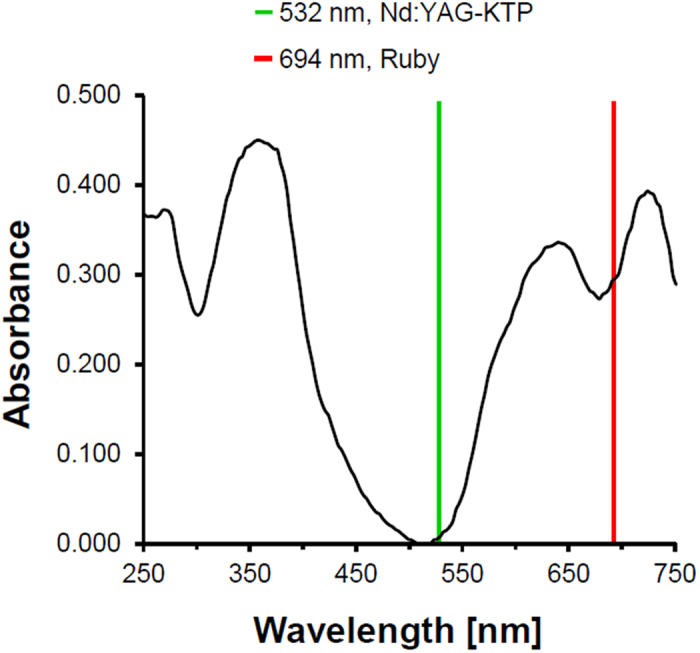
Absorption spectrum of pigment B15:3 at UV/VIS light. Wavelengths of ruby laser (694 nm) and the frequency doubled Nd:YAG (532 nm) are indicated by red and green lines, respectively.

By contrast, decomposition of pigment B15:3 was only low under Nd:YAG laser irradiation at both wavelengths applied (Fig. 3a–c). This can be explained in view of the absorption spectrum of pigment B15:3 which displays maxima in the visible spectrum at wavelengths between 550–800 nm (orange to red), thus being beyond the zone of coherent green light (532 nm) as emitted by the frequency doubled Nd:YAG laser (Fig. 4)^18^,^19^. With regard to its absorption behavior at 1,064 nm there is repeated proof in the literature that the pigment copper phthalocyanine does not undergo molecular vibrations in the near-infrared and hence is unable to absorb significant amounts of light in this part of the spectrum^20^,^21^.

The observed non-linear increases (saturation) in the levels of decomposition products formed upon laser irradiation (Fig. 3d) were—at least in part—due to the visible carbonization of the UV/VIS cuvettes occurring after multiple laser pulses (Fig. 3e). Similar to the whitening of the skin that occurs during tattoo laser removal, the light is being scattered at the damaged cuvette surface, resulting in a reduced transmission of laser energy to the pigment suspension. On the other hand, the color intensity of treated samples was slightly increasing after multiple laser pulses (Fig. 3e), an observation most likely due to the reduction of particle sizes that is known to result in higher color brilliance^22^. Declining particle sizes upon laser irradiation have been previously described by Ferguson *et al.*^13^.

### *In vitro* toxicity of HCN on human skin cells

**Figure 5:**
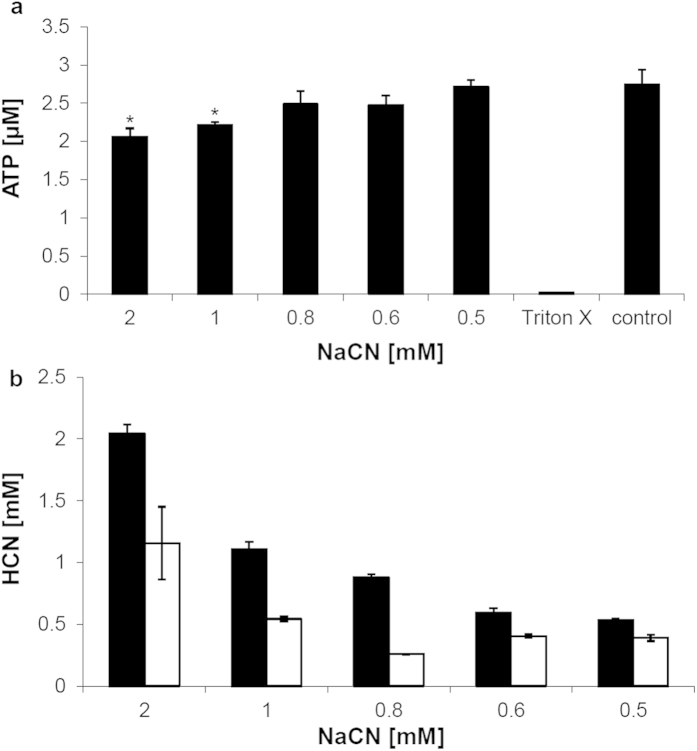
Toxicity of NaCN (respectively CN^–^ ions) in HaCaT cells *in vitro*. (**a**) ATP levels in cells treated with various doses of NaCN for 30 min in 96-well plates sealed with parafilm and compared to the control (that is, a 1:10 dilution of 5 × 10^−6^ N NaOH); Triton X was used as death control to verify depletion of ATP under toxic conditions. Values are displayed as mean ± SD (n = 4) for all groups except 0.6 mM NaCN (n = 3). Average ranks in control cells and cells treated with 1 mM and 2 mM NaCN were 6.5 and 2.5, respectively (Mann-Whitney U-Test, *p* = 0.029 two-tailed, *p < 0.05). Ranked, unequal variance *t*-test resulted in *p* = 0.005 and *p* = 0.013 for cells treated with 2 mM and 1 mM NaCN when compared to the controls, respectively. (**b**) HCN recovery from cell culture media before (black bars) and after 30 min of incubation at 37 °C (light bars). Prior to incubation, varying doses of NaCN were added to the media as indicated. Values are displayed as mean ± SD (n = 3).

We further looked into the cellular toxicity of HCN and observed significantly decreasing amounts of ATP at CN^–^ concentrations that lay in the range of the HCN levels detected after ruby laser irradiation of concentrated (>1.5 mg/ml) pigment dispersions (cf. Fig. 3a with Fig. 5a). Compromised cellular ATP synthesis results from the high binding affinity of CN^–^ to trivalent iron ions (i.e. Fe^3+^), which are part of the heme prosthetic group of cytochrome c oxidase in the mitochondrial electron transport chain and essentially required in the course of cellular ATP generation^23^,^24^. To assess cellular toxicity human HaCaT cells were exposed to increasing concentrations of sodium cyanide (NaCN) in 96-well plates (note: NaCN completely dissolves into Na^+^ and CN^–^ ions in aqueous solution). A significant decrease of cellular ATP levels was detected in cells treated with ≥1 mM CN^–^ when compared to controls (Fig. 5a). To verify the initial concentrations added and to rule out significant complexation of CN^–^ ions by Fe^3+^ in the media, samples were analyzed for HCN release upon acidification using DHS–GC/MS. Initial HCN concentrations quantified in the cell culture media at pH 7.4 nicely correlated to the added amounts of NaCN but were found subsequently depleted to 30–50% due to the outgassing of HCN during 30 min of incubation at 37 °C (Fig. 5b)^25^. The reasoning for the assessment of cellular toxicity after 30 min of incubation was based on both practical considerations in the performance of the assay as well as the kinetics of HCN outgassing vs. its possible tissue distribution. So, 30 min was considered an appropriate time-point mimicking the *in vivo* situation where local HCN concentrations would be diluted throughout tissue layers. The reduction of HCN concentrations down to 50% due to outgassing was another factor to be considered. Yet it seemed likely that this value found in aqueous solution *in vitro* could be also much lower in cornified skin *in vivo*.

## Discussion

Here we provide evidence for the suitability of Py–GC/MS analysis of tattoo pigments as reliable and predictive method for the generation and identification of chemical decomposition patterns that are likely to emerge upon laser irradiation of tattooed skin. Both q-switched ruby (694 nm) laser irradiation and pyrolysis-mediated fragmentation of the blue pigment copper phthalocyanine result in the same pattern of main cleavage products (i.e. benzene, BCN, BDCN, and HCN). Conversely, larger quantities of fragmentation products have neither been detected by applying the 1,064 nm nor the frequency-doubled 532 nm wavelength of an Nd:YAG laser. Since copper phthalocyanine is unable to absorb significant amounts of visible (green) light at 532 nm or infrared light at 1,064 nm^20^,^21^, it does not come as surprise that Nd:YAG lasers at these wavelengths reveal ineffective in clinical dermatology when it comes to the removal of blue colored tattoos^26^. While increasing concentrations of both benzene and HCN under ruby laser irradiation indicate the generation of temperatures in pigment deposits of at least 800 °C (cf. Fig. 1b,c), due to insufficient absorption Nd:YAG laser irradiation fails to generate temperatures high enough to cause particle fracturing^11^.

Among all of the compounds emerging upon ruby laser irradiation of copper phthalocyanine, HCN is of particular relevance due to its strong cellular toxicity. It has long been known as colorless, rapidly acting highly toxic gas (*bp* 26 °C)^27^,^28^. HCN was first prepared in 1782 by the Swedish chemist Carl Wilhelm Scheele from the dye “Prussian blue” (that is, “Berlin blue” or iron-III ferrocyanide: [Fe_4_[Fe(CN)_6_]_3_]) and therefore also named “prussic acid”^29^. Depending on the concentration inhaled, it can cause toxic effects and death from cardiac arrest within seconds^30^,^31^. The lethal dosage of HCN in most animal species is about 2 mg/kg body weight, and 50 ppm (0.005%) the concentration in air officially announced as “immediately dangerous to life or health” (IDLH) in humans^32^. From the toxicological point of view any possible emerging of HCN during laser removal of skin pigment deposits in tattooed individuals is thus to be regarded as health concern.

Here we demonstrate that the cell toxicity of HCN actually occurs in human skin cells *in vitro* at concentrations that lie in the range of the levels observed as result of ruby laser-mediated cleavage of copper phthalocyanine. According to the literature, minimum toxic concentrations of CN^–^ measured *in vitro* range from 100 μM to 10 mM depending on the various experimental set-ups that may differ in their extent of HCN outgassing^25^. Delhumeau and coworkers showed an inhibition of the cytochrome c oxidase at 250 μM potassium cyanide (KCN) in an activity test applying purified protein^33^. So, the levels of about 0.2–1.1 mM HCN that were dose dependently formed upon ruby laser treatment of 0.2–2.5 mg/ml pigment in suspension lie well within and beyond the concentration range of this compound shown to exert cellular toxicity. In this regard it is to be noted that the average tattooed individual usually features 100–300 cm^2^ of tattooed skin surface with pigment deposits that can range from 0.6 to 9.42 mg/cm^2^, depending on the experience of the tattooist^1^,^3^. (Note: The whole-body surface of an adult covers approximately 2 m^2^). Thus, the levels of chemical hazards such as HCN emerging upon laser removal of tattoos in human skin *in vivo* may greatly vary as function of tattoo size, pigment concentration and localization, irradiation dose (fluence) and wavelength applied. Still, levels as high as 1.1 mM HCN, measured in our study upon irradiation of 2.5 mg/ml pigment dispersion *in vitro*, would correspond to 29.7 μg HCN per ml of volume. Although the most common ways of cyanide poisoning are ingestion and inhalation, percutaneous absorption has also been repeatedly described for injured skin^34^,^35^, but also in non-injured skin^36^. Given an estimated level of 5 μg/ml cyanide in blood as lethal concentration^37^, local concentrations of about 30 μg/ml HCN that might be generated within tissue layers well supplied with blood vessels may trigger some concern, in particular when extremely large tattoos will be irradiated. Of course, the laser impact and its shattering efficiency on the pigments as well as the biokinetics of possibly emerging HCN in living skin tissue are currently unknown but certainly require urgent investigation in the future ahead.

Even though its concentrations measured upon ruby laser-induced pigment degradation were comparably low, the occurrence of the volatile aromatic hydrocarbon benzene further adds to these health concerns. According to U.S. EPA benzene is a “known human carcinogen by all routes of exposure”^38^. Induction of acute non-lymphocytic leukemia and other blood disorders such as aplastic anemia have been sufficiently supported only in highly exposed human workers via the inhalation route though.

Altogether our findings suggest that, in the course of laser removal, lightfast organic pigments such as copper phthalocyanine might be cleaved into toxic fragments in quantities likely to be capable of harming the integrity of patients’ skin locally and potentially also other tissues in the body systemically. Further studies applying real human skin tissue specimens *ex vivo* are necessary and will be conducted to shed further light on the issue of emerging HCN and benzene levels as consequence of the laser irradiation of blue colored pigment deposits in real human skin. Modifying interactions with cell or tissue constituents are well be conceivable and might lead to alterations of the decomposition pattern qualitatively (chemical composition) as well as quantitatively. In any case, however, in view of the data presented the consideration of possible cleavage products of tattoo pigments in the frame of future tattoo ink regulation becomes mandatory.

## Methods

### Chemicals and reagents

All chemicals, analytical standards and solvents used were of analytical or LC–MS grade. Potassium cyanide-^13^C-^15^N (isotope purity 99% and 98%, respectively), sodium cyanide (NaCN), sodium hydroxide (NaOH), benzene, benzene-d_6_ (isotope purity 97%), 1,2-benzene dicarbonitrile (BDCN), benzonitrile (BCN), benzylnitrile (BenzCN) and benzylalcohol (BenzOH) were obtained from Fluka/Sigma-Aldrich (Munich, Germany). Pigment B15:3 (C.I. 74160, m.w. 576.02; *mp* 600 °C) was kindly provided from Clariant as trade name product PV Fast Blue (Frankfurt, Germany). Ethyl acetate was purchased from Merck (Darmstadt, Germany).

**GC/MS.** If not described differently, the Agilent 7890A gas chromatograph was coupled to an Agilent 5975C inert XL MSD with Triple-Axis Detector (Agilent Technologies, Santa Clara, CA, USA). Ionization was induced by an inert electron impact (EI) ion source at 70 eV and helium (purity of 99.999%) from Air Liquide (Düsseldorf, Germany) was used as carrier gas.

### Pyrolysis–GC/MS of pigment B15:3

For Py–GC/MS analysis an HP-Plot/Q 30 m long column with a stationary phase of 0.32 mm thickness and 20 μm i.d. (Agilent Technologies, Santa Clara, CA, USA) was used. Small samples of pigment B15:3 were directly placed inside a glass tube which were then automatically inserted to the pyrolysis module at the thermal desorption unit (TDU) (both from Gerstel, Mühlheim, Germany) of the GC/MS inlet system. Pyrolysis was carried out at temperatures of 500–1,000 °C for 6 s. The temperature of the cold injection system (CIS), TDU and transfer line was maintained at 260 °C. The carrier gas flow rate was 1 ml/min using a split ratio of 1:30. The GC oven was kept at 50 °C for 2 min and afterwards ramped at 10 °C/min to 260 °C which was then held for 10 more minutes. The mass range was scanned in full scan mode from 10 to 550 m/z. Fragments were identified using a library of standards (US-NIST – National Institute of Standards and Technology – 2011 MS Library). Match and rematch for HCN, BCN and BDCN were above 900 on a scale to 999 being the best possible match. Data were analyzed using Enhanced ChemStation (E02.02.1431) from Agilent Technologies.

### Pigment dispersion and laser irradiation

Varying amounts of pigment B15:3 per ml ultrapure water (18.2 MΩ·cm at 25 °C) containing 5 × 10^−5^ N NaOH were weighted into an ND18 20 ml brown glass vial (Neolab, Heidelberg, Germany). A pH of ≥10 was ensured to prevent premature outgassing of emerging HCN during laser irradiation. For analysis of benzene, 1 mg/ml pigment B15:3 were weighted in ultrapure water without addition of NaOH. The dispersions were shaken vigorously by hand and then placed into a sonorex digitec ultrasonic water bath with 50/60 Hz (Bandelin Electronic, Berlin, Germany) for 60 min for further dispersion. The vials were shaken repeatedly to ensure best dispersion of pigment agglomerates which tend to stick at the walls or the caps of the vials. Then 200 μl of the dispersion was transferred into semi-micro UV/VIS cuvettes (Brand, Wertheim, Germany) and closed with polypropylene caps (Ratiolab, Dreieich, Germany). Samples were treated with multiple quantities of q-switched ruby (5 J/cm^2^, spot size 5 mm, pulse duration 20 ns, 694 nm, Sinon, WaveLight, Erlangen, Germany) or Nd:YAG (5 J/cm^2^, spot size 3 mm, pulse duration >20 ns, at 1,064 nm or 532 nm, Revlight SI, Cynosure, Westford, MA, USA) laser pulses and then placed on ice until further processing.

### DHS–GC/MS method for HCN and benzene quantification

NaCN standard substance was dissolved in aqueous 0.02 N NaOH to prevent outgassing. The basic solution was stored at −20 °C and freshly thawed right before usage. For further dilution, water containing 5 × 10^−5^ N NaOH was used to guarantee a pH value of 10. One ml of a standard solution or a 1:5 dilution of the laser sample was then transferred into an ND18 20 ml brown glass vial (Neolab, Heidelberg, Germany) and 10 μl of a stock solution of 100 μg/ml K^13^C^15^N were added as internal standard. The cap on top of the vial was screwed firmly and 50 μl of 30% phosphoric acid was then added through the septum using a 100 μl Hamilton syringe (Neolab, Heidelberg, Germany). The last step guaranteed protonation of dissolved cyanide ions to form gaseous HCN. Samples designated for benzene quantification were directly processed after laser irradiation. To this end, 200 μl of the irradiated pigment dispersion was transferred into an ND18 brown glass vial (20 ml) containing 800 μl of 5 × 10^−5^ N NaOH and screwed immediately. Benzene-d_6_ was added as internal standard through the septum using a Hamilton syringe to achieve a final concentration of 5ng/ml. Prior to the laser experiments the linearity of benzene quantification was confirmed within the range of 1 to 500 ng/ml, and a one-point calibration for the test samples was carried out. For subsequent DHS–GC/MS analysis of HCN and benzene an HP-Plot/Q system (Agilent Technologies, Santa Clara, CA, USA) was used (cf. above). Samples were incubated for 3 min at 30 °C before trapping the gas phase on a Carbopack B+X/Carboxen-1000 desorption liner (Gerstel, Mühlheim, Germany) with agitation. The trapping of samples was carried out with a volume of 100 ml N_2_ gas and a flow rate of 50 ml/min. Analytes were desorbed from the liner in a TDU (Gerstel, Mühlheim, Germany) and cryofocused in a CIS at −50 °C. After 12 s the temperature of the CIS was increased to 40 °C and held for 5.5 min followed by a second increase to 240 °C which was held for additionally 5 min. TDU and transfer line were kept constant at 260 °C. Injections were carried out in splitless mode. The initial oven temperature of 40 °C was held for 0.5 min and then heated by 10 °C/min up to 260 °C which was held for 10 more minutes. The mass range was scanned in the scan/single ion mode (SIM) from 10 to 350 m/z with acquisition ions 27, 28, 78 and 84 with a dwell time of 40 ms, respectively.

Based on the overall amount of HCN, BCN and BDCN measured upon laser irradiation the percent fraction of pigment B15:3 destroyed has been calculated in relation to the maximum possible release of CN residues per molecule pigment (that is, 8 × CN; cf. Fig. 2).

### GCxGC-ToF–MS

The Pegasus 4D GCxGC-ToF–MS system (Leco, Mönchengladbach, Germany) was used for analysis of the extracts of the laser experiments. Rxi®-5Sil MS (20 m, 0.25 mm, 0.25 μm i.d.) and Rxi®-17Sil MS (1 m, 0.18 mm, 0.18 μm i.d.) columns (Restek, PA, USA) were arranged as first and second dimension columns, respectively. The initial oven temperature was set to 70 °C and remained for 1 min followed by the first ramp with 15 °C/min to 120 °C, the second ramp with 8 °C/min to 150 °C, and the third ramp with 25 °C/min to 330 °C, which was finally held for 4 min. Secondary oven temperature and modulator oven temperature were 5 °C and 15 °C higher relative to the oven temperature, respectively. The modulator period was 3.5 s with a hot pulse time of 1 s and a cool time period between 0.75 s and 13 min of the GC program and elongated to 4 s with a hot pulse time of 1.5 s till the end of the run. The chiller was cooled to −80 °C. Front inlet flow was 1 ml/min. The temperatures of the ion source and transfer line were set to 250 °C and 295 °C, respectively. Mass spectra were collected with an acquisition rate of 200 Hz in a mass rage of 35–500 m/z. BCN and BDCN were quantified by using BenzCN and BenzOH as internal standards. To this end, 4 μl of a mixture containing equal parts of BenzOH (2 μg/ml) and BenzCN (0.5 μg/ml) were added to 196 μl sample and capped in a 2 ml glass vial. After extraction with 200 μl ethyl acetate for 1 hour while shaking, 1.5 μl of the extract were analyzed using GCxGC-ToF–MS.

### Cytotoxicity assay

HaCaT cells were grown in Dulbecco’s modified Eagle medium (DMEM) with 1 g/l glucose plus 5% fetal bovine serum (FBS) supplemented with penicillin/streptomycin (10,000 U/ml; 10 mg/ml) and 5 ml of L-glutamine (200 mM) until they reached 50% density in 96-well plates. All reagents were purchased from PAH Biotech (Aidenbach, Germany). Prior to the assay, cells were washed with phosphate buffered saline (PBS) and DMEM without FBS was added to the wells. From a stock solution of 1 mg/ml NaCN dissolved in 5 × 10^−6^ N NaOH aliquots of no more than 10 μl were directly transferred into the well with a pipette (dilution of 1:10). As controls, 10 μl of 5 × 10^−6^ N NaOH without NaCN or 2 μl of 1:10 Triton X were added to the cells. Subsequently 96-well plates were sealed with parafilm (Pechiney Plastic Packaging Inc., Washington, NJ, USA) and incubated for 30 minutes.

For the determination of ATP levels cells were treated as follows: Cultures were rinsed with 100 μl PBS first. Then 50 μl of lysis buffer (ATP Bioluminescence Assay Kit HS II; Roche Diagnostics, Mannheim, Germany) were added to the wells and further incubated for 5 min at room temperature followed by 2 min at 99 °C. Afterwards, 50 μl PBS were added and plates were frozen and stored at −20 °C until further processing. Cell debris was separated by centrifugation at 5,000 g for 10 min (Thermo Scientific Heareus Multifuge 1s-r, MA, USA) and 40 μl supernatant was transferred into a white 96-well plate. Luminescence was analyzed without filters for 10 s in each well after automated injection of 40 μl luciferase reagent.

### UV/VIS Absorbance

Spectral absorbance of pigment B15:3 (2 μl of an 0.2 mg/ml aqueous pigment dispersion) was measured using the NanoDrop® 1000 spectrophotometer (Peqlab, Erlangen, Germany) according to manufacturer’s instructions.

### Statistics and calculations

The results are presented as the mean values (mean ± standard deviation (SD)). Data analyses were performed using IBM SPSS Statistics (Version 21, Armonk, NY, USA). Due to small sample sizes data were regarded as non-normally distributed and unequal in variances. Statistical significance was therefore tested using the non-parametrical Mann-Whitney U-Test for two independent samples. The exact 2-tailed significance was used for group comparison. Additionally, a ranked, unequal variance *t*-test was carried out. A *p*-value of <0.05 was considered significant and marked with an asterisk.

## Additional Information

**How to cite this article**: Schreiver, I. *et al.* Formation of highly toxic hydrogen cyanide upon ruby laser irradiation of the tattoo pigment phthalocyanine blue. *Sci. Rep.* **5**, 12915; doi: 10.1038/srep12915 (2015).
